# A Robust Static Decoupling Algorithm for 3-Axis Force Sensors Based on Coupling Error Model and *ε*-SVR

**DOI:** 10.3390/s121114537

**Published:** 2012-10-29

**Authors:** Junqing Ma, Aiguo Song, Jing Xiao

**Affiliations:** 1 Jiangsu Key Lab of Remote Measurement and Control, School of Instrument Science and Engineering, Southeast University, Nanjing 210096, China; E-Mail: mjqseu@gmail.com; 2 IMI Lab, Department of Computer Science, The University of North Carolina at Charlotte, Charlotte, NC 28223, USA; E-Mail: xiao@uncc.edu

**Keywords:** force sensors, coupling errors, decoupling, support vector regression (SVR)

## Abstract

Coupling errors are major threats to the accuracy of 3-axis force sensors. Design of decoupling algorithms is a challenging topic due to the uncertainty of coupling errors. The conventional nonlinear decoupling algorithms by a standard Neural Network (NN) are sometimes unstable due to overfitting. In order to avoid overfitting and minimize the negative effect of random noises and gross errors in calibration data, we propose a novel nonlinear static decoupling algorithm based on the establishment of a coupling error model. Instead of regarding the whole system as a black box in conventional algorithm, the coupling error model is designed by the principle of coupling errors, in which the nonlinear relationships between forces and coupling errors in each dimension are calculated separately. Six separate Support Vector Regressions (SVRs) are employed for their ability to perform adaptive, nonlinear data fitting. The decoupling performance of the proposed algorithm is compared with the conventional method by utilizing obtained data from the static calibration experiment of a 3-axis force sensor. Experimental results show that the proposed decoupling algorithm gives more robust performance with high efficiency and decoupling accuracy, and can thus be potentially applied to the decoupling application of 3-axis force sensors.

## 1. Introduction

Force sensing is crucial for on-line perception and feedback in interactions between intelligent robotic manipulators and environments. Multi-axis force sensors are used to perceive generalized force information and convert input force signals to voltage signals [1]. They are usually mounted on the tips of robot arms for automatic contact recognition, motion planning, and force control tasks [2–4]. Force sensors are widely used in assembly robots, polishing robots, rehabilitation robots, *etc.* [5–7].

The accuracy of multi-axis force sensors has a great impact on force-perception based tasks with high precision requirements. This motivates the need to improve measurement precision. For a multi-axis force sensor, a key issue is that input force in one dimension may affect not only output of this dimension but also those of the other dimensions. Errors caused in this way, called the *coupling errors*, are major threats to the accuracy of multi-axis force sensors. Coupling errors occur for various reasons, such as mechanical structures, limitation of machining accuracy, transverse effect of strain gauges, *etc.* Song *et al.* in [8] developed a self-decoupled 4-axis force/torque sensor to reduce coupling errors by improving hardware design. However, in most cases, it is costly and sometimes infeasible to avoid coupling errors by improving the hardware design and machining accuracy. Decoupling algorithms are always used to reduce coupling errors.

The common static decoupling algorithm calculates the pseudo-inverse matrix of calibration data based on the Least Square Method (LSM) [9–11]. This algorithm is based on the assumption that relationships between input forces and output voltages in all dimensions are linear. Afterwards, the transfer matrix between input forces and output voltages are calculated. The obtained transfer matrix is called *calibration matrix*. Voyles *et al.* in [12] proposed a fast linear decoupling technique called *shape from motion* in which the motion of the force vector and the calibration matrix are simultaneously extracted by singular value decomposition from raw sensor signals. Cao *et al.* in [13] explored a linear static decoupling method using an NN to increase the accuracy of decoupling. However, large amounts of experiment data indicate the nonlinearity in relationships between forces and coupling errors. Thus, the precision of linear decoupling algorithms is limited and unsatisfactory. Other approaches [14,15] employed a feed-forward NN with back propagation (BP) training algorithms to realize the nonlinear Multiple Input Multiple Output (MIMO) mapping of a multi-axis force sensor. In [15], the authors also used a standard radial basis function (RBF) NN for decoupling. Engineering applications show that decoupling algorithms with a standard NN model can sometimes reduce coupling error significantly, but sometimes generate worse results than without decoupling due to overfitting.

Support Vector Machine (SVM) is a powerful candidate for decoupling algorithms due to its ability to perform adaptive and nonlinear data fitting. SVM starts from solving problems of classification. With the introduction of Vapnik's *ε*-insensitive loss function, it also extends to be a regression prediction tool that uses machine learning theory to maximize predictive accuracy while not subject to local minimal and overfitting [16]. Support Vector Machine for regression, called *Support Vector Regression*, gradually becomes a powerful tool for nonlinear correcting and compensation in the field of sensors. Guo *et al.* in [17] established a model based on SVR to correct the nonlinear error of photoelectric displacement sensor. Wang in [18] used SVR to make nonlinear estimation and temperature compensation of capacitor pressure sensors.

The design of decoupling algorithms of 3-axis force sensors presents several challenges. First, a 3-axis force sensor is usually used in on-line force perception tasks. This requires the sensor to show quick response to variations of input forces. Thus, the decoupling algorithms should have high time efficiency. Second, inevitable noises in calibration data may result in overfitting such that decoupling precision will be reduced. Also, due to improper operations of laboratory technicians or environment disturbances, occasionally there may be gross errors in calibration data. Gross errors are outliers that strongly deviate from the majority of experiment data. Gross errors may result in unexpected decoupling results such that decoupling precision will be reduced. A reliable decoupling algorithm should be designed to minimize the negative effects of noises and gross errors. Third, because the hardware causes of coupling errors such as the mechanical structures and the limitations of machining accuracy are complex and uncertain, it is difficult to model the coupling errors by polynomials. Thus, a decoupling algorithm should have a high generalization ability.

Motivated by the above challenges, this paper proposes a precise and fast decoupling algorithm with high reliability. Instead of referring to the whole sensor system as a black box using one standard NN [14,15], the proposed decoupling algorithm is designed using the principle of coupling errors, in which the relationships between each input and output are mapped separately in the proposed coupling error model to make the algorithm more reliable. The proposed coupling error model consists of six SVRs and three linear fitting functions, which is more conformable to calibration data structure. Our method is compared with the standard NN method, as they are applied to the same data from a calibration experiment, and our method gives better reliability and higher efficiency.

The remainder of the paper is organized as follows. Section 2 introduces a novel model of coupling error and its notations. *ε*-SVR is described as the nonlinear approximation tool of the model. A decoupling process based on the model is proposed. Section 3 briefly describes the principle of a 3-axis sensor designed in our lab, the calibration experiment process and the structure of the calibration data. Section 4 discusses the implementation details of the decoupling method using a set of experimental data obtained from a calibration experiment. In order to demonstrate that our decoupling algorithm is robust to gross errors, gross errors were artificially introduced into the calibration data and the decoupling accuracy with the gross errors were calculated. Finally, in Section 5 some conclusions are provided.

## 2. Nonlinear Static Decoupling

### 2.1. Coupling Error Model and Notations

We first establish an appropriate coupling error model to capture the relationships between input forces and corresponding coupling errors. In the model, the input forces and output voltages of a 3-axis force sensor in X, Y, Z directions are defined as *f_x_*, *f_y_*, *f_z_* and *u_x_*, *u_y_*, *u_z_*, respectively.

For each dimension, output voltages are partitioned into two categories. One category includes the voltages corresponding to input forces in the same dimension, called *prime voltages*. The other category includes the voltages corresponding to the input forces in the other two dimensions, called *coupling errors*. We use *u_xx_*, *u_yy_*, *u_zz_* to denote prime voltages and *e_x_*, *e_y_*, *e_z_* to denote coupling errors in X, Y, Z directions, respectively. Prime voltages account for the majority of output voltages. Next, coupling errors are separated into two *coupling error elements* caused by input forces of different dimensions. Let (*e_xy_*, *e_xz_*) represent the coupling error element in X direction, where *e_xy_* refers to the coupling error element caused by *f_y_*, and *e_xz_* refers to the coupling error element caused by *f_z_*. Similarly, we split the coupling error in Y direction into *e_yx_* and *e_yz_*, and split the coupling error in Z direction into *e_zx_* and *e_zy_*. We can get:

(1) $$\{\begin{array}{c} u_{x}=u_{\text{xx}}+e_{\text{xy}}+e_{xz}\\ u_{y}=u_{yy}+e_{\text{yx}}+e_{\text{yz}}\\ u_{z}=u_{zz}+e_{\text{zx}}+e_{\text{zy}} \end{array}$$

Based on the observation of calibration data of multi-axis force sensors in our lab, we make the following assumptions about coupling errors.

1. The relationship between the prime force and the prime voltage in every dimension is linear;
2. Relationships between disturbing force and their corresponding coupling error elements in every dimension are nonlinear;
3. The above relationships are independent and time-invariant.

**Figure 1.**
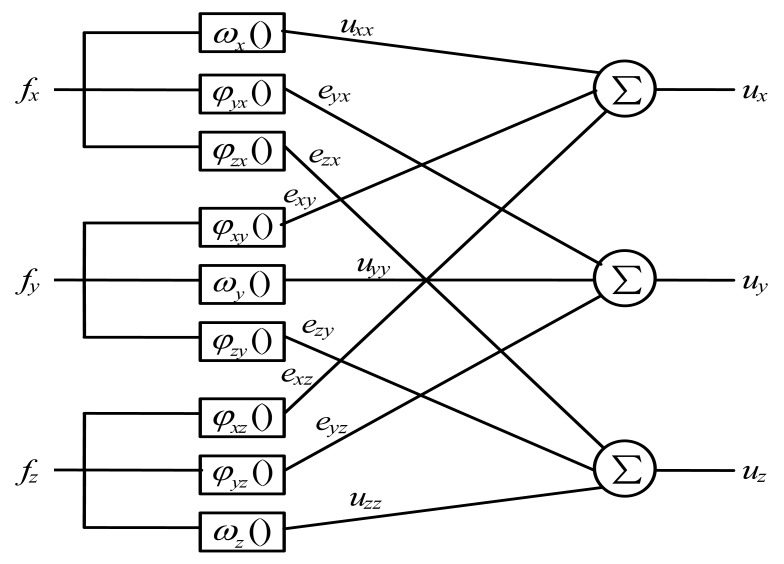
A coupling error model of 3-axis force sensors.

From the above assumptions and the principle of superposition for stress, we propose a coupling error model as shown in Figure 1.

In Figure 1, there are three layers in the coupling error model: the input layer, the output layer, and the middle layer. Nine nodes in the middle layer are parallel and separated from each other. Functions *ω_x_*(), *ω_y_*() and *ω_z_*(), are *non-coupling functions*, linearly relating prime force to prime voltage in each dimension as shown in Equation (2).

(2) $$\{\begin{array}{c} u_{\text{xx}}=\omega_{x}(f_{x})=k_{\text{xx}}\cdot f_{x}\\ u_{yy}=\omega_{y}(f_{y})=k_{yy}\cdot f_{y}\\ u_{zz}=\omega_{z}(f_{z})=k_{zz}\cdot f_{z} \end{array}$$

In calibration experiments, only one-dimensional force is applied to a 3-axis force sensor each time, while the output voltages of all directions are recorded simultaneously. Detailed calibration experiment process will be described in Section 3. Consequently, as for calibration data, no coupling error exists in the output voltage corresponding to the direction of the input force and no prime force exists in the output voltages of other directions. In other words, the output voltage equals the prime voltage when the direction of the output voltage is the same as the direction of the input force, and the output voltage equals the corresponding coupling error elements when the direction of the output voltage is different from the direction of the input force. For instance, during calibration experiment of X direction, a set of standard *f_x_*_(_*_c_*_)_ are applied to the force sensor while *f_y_*_(_*_c_*_)_ and *f_z_*_(_*_c_*_)_ remain zero (the subscript *c* represents “calibration data”). Thus we can get *u_x_*_(_*_c_*_)_ = *u_xx_*_(_*_c_*_)_, *u_y_*_(_*_c_*_)_ = *e_yx_*_(_*_c_*_)_, *u_z_*_(_*_c_*_)_ = *e_zx_*_(_*_c_*_)_. Similarly, for calibration data in Y direction, *u_x_*_(_*_c_*_)_ = *e_xy_*_(_*_c_*_)_, *u_y_*_(_*_c_*_)_ = *u_yy_*_(_*_c_*_)_, *u_z_*_(_*_c_*_)_ = *e_zy_*_(_*_c_*_)_; for calibration data in Z direction, *u_z_*_(_*_c_*_)_ = *e_xz_*_(_*_c_*_)_, *u_y_*_(_*_c_*_)_ = *e_yz_*_(_*_c_*_)_, *u_z_*_(_*_c_*_)_ = *u_zz_*_(_*_c_*_)_.Thus, coefficients *k_xx_*, *k_yy_*, and *k_zz_* in Equation (2) can be calculated by linear fitting of prime forces and prime voltages of calibration data using LSM.

Functions respecting nonlinear relationships between disturbing forces and corresponding coupling error elements in every dimension are *φ_yx_*(), *φ_zx_*(), *φ_xy_*(), *φ_zy_*(), *φ_xz_*(), *φ_yz_*(), called *coupling functions* as shown in Equation (3).

(3) $$\{\begin{array}{c} e_{\text{yx}}=\varphi_{\text{yx}}(f_{x});e_{\text{zx}}=\varphi_{\text{zx}}(f_{x});\\ e_{\text{xy}}=\varphi_{\text{xy}}(f_{y});e_{\text{zy}}=\varphi_{\text{zy}}(f_{y});\\ e_{xz}=\varphi_{xz}(f_{z});e_{\text{yz}}=\varphi_{\text{yz}}(f_{z}); \end{array}$$

Then, *corrected coupling functions χ*() can be obtained from Equations (2) and (3):

(4) $$\{\begin{array}{c} e_{\text{yx}}=\varphi_{\text{yx}}(f_{x})=\varphi_{\text{yx}}\left(\omega_{x}^{-1}(u_{\text{xx}})\right)=\chi_{\text{yx}}(u_{\text{xx}})\\ e_{\text{zx}}=\varphi_{\text{zx}}(f_{x})=\varphi_{\text{zx}}\left(\omega_{x}^{-1}(u_{\text{xx}})\right)=\chi_{\text{zx}}(u_{\text{xx}})\\ e_{\text{xy}}=\varphi_{\text{xy}}(f_{y})=\varphi_{\text{xy}}\left(\omega_{y}^{-1}(u_{yy})\right)=\chi_{\text{xy}}(u_{yy})\\ e_{\text{zy}}=\varphi_{\text{zy}}(f_{y})=\varphi_{\text{zy}}\left(\omega_{y}^{-1}(u_{yy})\right)=\chi_{\text{zy}}(u_{yy})\\ e_{xz}=\varphi_{xz}(f_{z})=\varphi_{xz}\left(\omega_{z}^{-1}(u_{zz})\right)=\chi_{xz}(u_{zz})\\ e_{\text{yz}}=\varphi_{\text{yz}}(f_{z})=\varphi_{\text{yz}}\left(\omega_{z}^{-1}(u_{zz})\right)=\chi_{\text{yz}}(u_{zz}) \end{array}$$

During a decoupling process of an actual force perception task, one can hardly obtain the exact value of prime voltages *u_xx_*, *u_yy_*, *u_zz_* for Equation (4), because the data obtained in a force perception task (called *task data*) are different from calibration data. The input forces in all directions are always non-zero, so the output voltages in all directions contain coupling errors and the output voltages no longer equal the prime voltages. However, a large number of experiment data show that, in most cases, coupling errors take up less than 5% of the full scale (F.S.) output voltages and the absolute values of the slopes of the corrected coupling functions are no more than 0.05. Thus, the output voltages of task data *u_x_*_(_*_t_*_)_, *u_y_*_(_*_t_*_)_, *u_z_*_(_*_t_*_)_ approximately equal the prime voltages *u_xx_*_(_*_t_*_)_, *u_yy_*_(_*_t_*_)_, *u_zz_*_(_*_t_*_)_ (the subscript *t* represents “task data”), and can be used as substitutions of the prime voltages *u_x_*_(_*_t_*_)_, *u_y_*_(_*_t_*_)_, *u_z_*_(_*_t_*_)_ as independent variables of Equation (4). This approximate substitution may induce second-order coupling errors. Take *e_yx_* for example: the measured coupling error element *e_yx_*_(_*_m_*_)_ = *χ_yx_*(*u_x_*_(_*_t_*_)_) is calculated instead of the actual coupling error element *e_yx_*_(_*_a_*_)_ = *χ_yx_*(*u_xx_*_(_*_t_*_)_)(the subscript *m* represents “measured”; the subscript *a* represents “actual”). The second-order coupling error *E_e_yx__* can be expressed in Equation (5),

(5) $$E_{e_{\text{yx}}}=\frac{\left|e_{yx(m)}-e_{yx(a)}\right|}{u_{x(F.S.)}}=\frac{\left|\chi_{\text{yx}}(u_{x(t)})-\chi_{\text{yx}}(u_{xx(t)})\right|}{u_{x(F.S.)}}$$

where *u_x_*_(_*_F.S._*_)_ represents the full scale value of output voltages in X direction.

According to the Lagrange mean value theorem, ∃*ξ* ∈ (*u_xx_*_(_*_t_*_)_, *u_x_*_(_*_t_*_)_) such that

(6) $$E_{e_{\text{yx}}}=\frac{\left|\chi_{\text{yx}}^{\prime }(\xi )\right|\left|(u_{x(t)}-u_{xx(t)})\right|}{u_{x(F.S.)}}\leq \frac{0.05\cdot (5\%u_{x(F.S.)})}{u_{x(F.S.)}}=0.25\%$$

As a result, the rate of second-order coupling errors is less than 0.25% and can be neglected in most industrial applications.

We choose *ε*-SVR for nonlinear approximations for the corrected coupling functions, which is much less likely to subject to overfitting problems. Also, the fitting processes of those functions are independent from one dimension to another, hence the fitting result of one dimension does not affect that of another dimensions.

### 2.2. Approximation of Corrected Coupling Functions Using *ε*-SVR

Six *ε*-SVRs are utilized to approximate six corrected coupling functions for its generalization ability. *ε*-SVR learns the relationship between the input (*i.e.*, prime voltages) and the output (*i.e.*, corresponding coupling error elements) by adjusting the structure and parameters of a flexible model directly from training data to minimize the prediction error.

The basic idea for the case of nonlinear regression by *ε*-SVR is to project the input space *x_i_* to a higher dimensional feature space by a map Φ. Then, the *ε*-SVR defines a linear prediction model over the mapped samples in the feature space. A nonlinear function is learned by this model while the capacity of the model is controlled by a parameter that does not depend on the dimensionality of the space [16]. As calculation with the map Φ can easily become computationally infeasible (because it is too complex), a kernel function *k* is introduced as the dot product of Φ, as expressed in Equation (7).

(7) $$<\Phi (x_{i}),\Phi (x_{j})>=k(x_{i},x_{j})$$

Typical kernels include the linear $k(x_{i},x_{j})=x_{i}^{T}=x_{j}$, the polynomial $k(x_{i},x_{j})={\left(x_{i}^{T}x_{j}+1\right)}^{d}$, and the Gaussian Function(RBF) *k*(*x_i_*, *x_j_*) = exp(−‖*x_i_* − *x_j_*‖^2^/2*δ*^2^). The RBF kernel is most frequently used and it is also the one used in our implementation.

**Figure 2.**
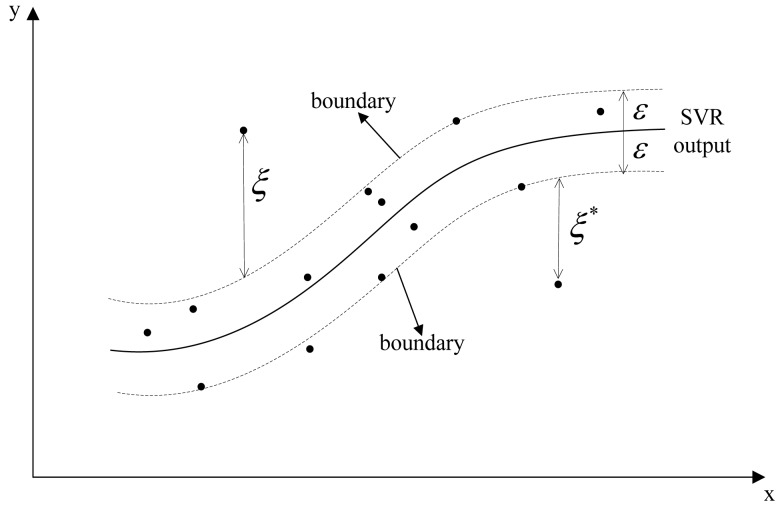
Graphic description of the *ε*-SVR model for a linear case.

Briefly, the *ε*-SVR is to solve a convex optimization problem:

(8a) $$\begin{array}{cc} \text{minimize} & \frac{1}{2}{\left\|w\right\|}^{2}+C\sum_{i=1}^{Q}\left(\xi_{i}+\xi_{i}^{*}\right) \end{array}$$

(8b) $$\begin{array}{cc} \text{subject}\;\text{to} & \{\begin{array}{l} y_{i}-w\Phi (x_{i})-b\leq ɛ+\xi_{i}\\ w\Phi (x_{i})+b-y_{i}\leq ɛ+\xi_{i}^{*}\\ \xi_{i},\xi_{i}^{*}\geq 0 \end{array} \end{array}$$

where *i* = 1,2, … , *Q* are training data points, the parameter *w* indicates the flatness of regression function *f* due to the fact that kernels can be associated with flatness properties via regularization operators [19]. *ξ_i_*, $\xi_{i}^{*}$ are slack variables introduced by Vapnik's *ε*-insensitive loss function [20], in which errors up to *ε* are not penalized, and all further deviations will incur a linear penalization [21]. *C* > 0 is the regularization parameter determining the trade-off between the flatness of regression function *f* $\left(i.e.,\frac{1}{2}{\left\|w\right\|}^{2}\right)$ and the total tolerance on deviations larger than *ε* $\left(i.e.,\sum_{i=1}^{Q}(\xi_{i}+\xi_{i}^{*})\right)$. A graphical description of *ε*-SVR model is shown in Figure 2. Points on the boundaries and outside the boundaries are called *Support Vectors*.

To solve the optimization problem of Equation (8), a Lagrange function is constructed by introducing Lagrange multipliers *a_i_* and $a_{i}^{*}$. Partial derivatives of the Lagrange function with respect to the primal variables (*w*, *b*, *ξ_i_*, $\xi_{i}^{*}$) are calculated. Finally, the dual optimization problem can be derived by partial derivation of the Lagrange function and taking into account that $\xi_{i}\xi_{i}^{*}=0$:

(9a) $$\begin{array}{cc} \text{maximize} & -\frac{1}{2}\sum_{i,j=1}^{Q}\left(a_{i}-a_{i}^{*}\right)\left(a_{j}-a_{j}^{*}\right)k\left(x_{i}x_{j}\right)-\varepsilon \sum_{i=1}^{Q}\left(a_{i}+a_{i}^{*}\right)+\sum_{i=1}^{Q}\left(a_{i}-a_{i}*\right) \end{array}$$

(9b) $$\begin{array}{cccc} \text{subject}\;\text{to} & \sum_{i=1}^{Q}\left(a_{i}-a_{i}^{*}\right)=0 & \text{and} & a_{i},a_{i}^{*}\varepsilon \left[0,C\right] \end{array}$$

Note that Support Vectors correspond to training data whose Lagrange multipliers *a_i_*'s are non-zero in Equation (9). Training data for which *a_i_* = *C* are called Bounded Support Vectors, and they are located on the two boundaries in Figure 2. Training data with 0 < *a_i_* < *C* are called Free Support Vectors and they are outside the boundaries [22]. Only Support Vectors contribute to the penalization in Equation (8a). Equation (9) can be solved by optimization algorithms such as Sequential Minimal Optimal, Chunking and Decomposing. The Sequential Minimal Optimal [23] is most frequently used due to its fast calculation speed. Solving the quadratic problems of Equation (9) yields the final solution of *ε*-SVR:

(10) $$f(x)=\sum_{i=1}^{Q}(a_{i}-a_{i}^{*})k(x_{i},x)+b$$

It is shown that *ε*-SVR has properties of robustness to noises and gross errors even though the gross errors are part of the set of Support Vectors [24,25]. This is because the Lagrange Multipliers solved in the objective function of Equation (9) are upper bounded by the constraint of Equation (9). All Support Vectors, including the gross errors, will have Lagrange Multipliers with absolute value no more than the upper bound *C*. From aspect of Equation (8), as *C* searches for trade-off between flatness and empirical risk, if necessary, *ε*-SVR will sacrifice the tolerance to get a good flatness. *ε*-SVR also has many other advantages such as global minima and reduced likelihood of overfitting.

### 2.3. Decoupling Process

The whole decoupling process consists of two stages. The first stage is for establishment of the coupling error functions. The second stage is in an actual on-line force perception task. During the task, the output voltages of all directions are obtained and the coupling error model is utilized to eliminate coupling errors and calculate measured forces. Our decoupling process is described as follows:

1. Conduct the static calibration experiment of a 3-axis force sensor and get the calibration data, which contain sets of input forces and output voltages (*f_x_*_(_*_c_*_)_, *f_y_*_(_*_c_*_)_, *f_z_*_(_*_c_*_)_ and *u_x_*_(_*_c_*_)_, *u_y_*_(_*_c_*_)_, *u_z_*_(_*_c_*_)_).
2. Do linear fitting of *f_x_*_(_*_c_*_)_ and *u_x_*_(_*_c_*_)_ from calibration data in X direction using LSM, to get the slope *k_xx_* in Equation (2). In the same way, get coefficients *k_yy_* and *k_zz_* in Equation (2) from the calibration data in Y direction and Z direction respectively. In this way, three non-coupling functions *ω_x_*(),*ω_y_*() and *ω_z_*() and three inverse functions of the non-coupling functions $\omega_{x}^{-1}()$, $\omega_{y}^{-1}()$ and $\omega_{z}^{-1}()$ are obtained.
3. Do nonlinear function approximation for *u_x_*_(_*_c_*_)_ and *u_y_*_(_*_c_*_)_ from the calibration data in X direction by *ε*-SVR to get the corrected coupling function *χ_yx_*(), do nonlinear function approximation for *u_x_*_(_*_c_*_)_ and *u_z_*_(_*_c_*_)_ from the calibration data in X direction by *ε*-SVR to get the corrected coupling function *χ_zx_*().
4. Calculate the corrected coupling functions *χ_xy_*() and *χ_zy_*() from calibration data in Y direction and *χ_xz_*() and *χ_yz_*() from calibration data in Z direction respectively in the same way as in step 3.

The above steps establish the entire coupling error model. The following steps are designed for decoupling based on the model using a 3-axis force sensor in an actual force perception task.

- Obtain task data, namely, a set of output voltages (*u_x_*_(_*_t_*_)_, *u_y_*_(_*_t_*_)_, *u_z_*_(_*_t_*_)_) of a 3-axis force sensor, during an actual force perception task. Compute each coupling error elements of measured data using the corrected coupling functions.
- Subtract the coupling errors from the output voltages to get prime voltages as shown in Equation (1). Then calculate prime forces of measured data, namely, the measured three dimensional forces by $\omega_{x}^{-1}()$, $\omega_{y}^{-1}()$ and $\omega_{z}^{-1}()$. The measured forces *f_x_*_(_*_m_*_)_,*f_y_*_(_*_m_*_)_,*f_z_*_(_*_m_*_)_ can also be expressed in Equation (11).
  (11) $$\{\begin{array}{l} f_{x(m)}=w_{x}^{-1}[u_{x(t)}-\chi_{\text{xy}}(u_{y(t)})-\chi_{xz}(u_{z(t)})]\\ f_{y(m)}=w_{y}^{-1}[u_{y(t)}-\chi_{\text{yx}}(u_{x(t)})-\chi_{\text{yz}}(u_{z(t)})]\\ f_{z(m)}=w_{z}^{-1}[u_{z(t)}-\chi_{\text{zx}}(u_{x(t)})-\chi_{\text{zy}}(u_{y(t)})] \end{array}$$

**Figure 3.**
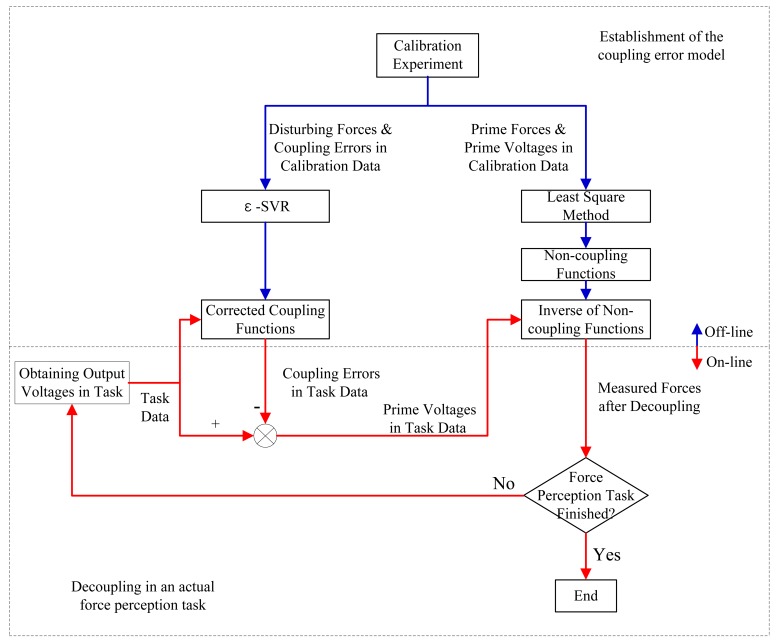
The flow chart of the whole decoupling process.

Figure 3 shows the flow chart of the whole decoupling process. The blue arrows indicate off-line process of the establishment of the coupling error model with calibration data. The red arrows indicate on-line process of decoupling in an actual force perception task. Note that the coupling error model and decoupling process are proposed with respect to a predefined frame based on the structural characteristics of the sensor. During an actual force perception task, if the orientation of the predefined frame is different from that of the reference frame used for force perception, the measured forces after decoupling with respect to the predefined frame should be transformed by a rotation matrix in order to align with the reference frame.

## 3. Calibration Experiment

As an example 3-axis force sensor, we consider a 3-axis force sensor designed and fabricated in our lab. The force measurement range in both X direction and Y direction is −100 N to +100 N, and the force measurement range in Z direction is −150 N to 0 N. The corresponding output voltages range in X direction and Y direction is −1.4 V to +1.4 V, and the output voltages range in Z direction is −1.2 V to +1.2 V.

The key component of the 3-axis force sensor is composed of a cross-beam elastic body with strain gauges, Wheatstone bridges, and a sampling circuit [26]. The principle of the 3-axis force sensor can be briefly described as follows: under the effect of loading forces, the cross-beam elastic body begins to deform measurably. Strain gauges that are pasted firmly on the surface of the cross-beam elastic body can sense the strains and transform them to changes in resistance. Resistance changes of strain gauges are measured by Wheatstone bridges and transformed to voltages. Finally, the sampling circuit amplifies the output voltages of Wheatstone bridges and transmits the amplified voltage signals to the robot controller.

**Figure 4.**
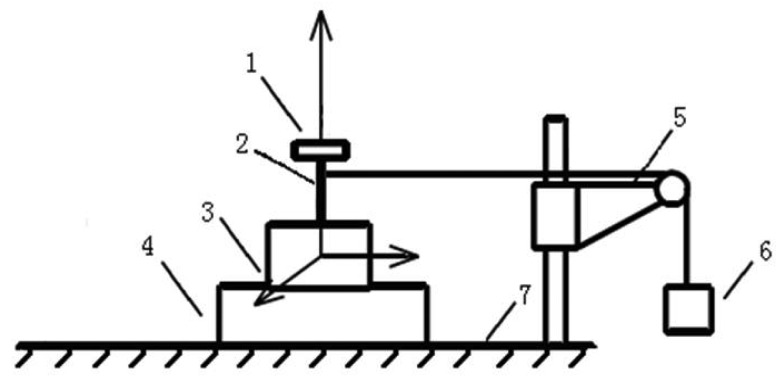
Diagram of static calibration experiment setup: (1) loading plate, (2) calibration shaft, (3) three-axis force sensor, (4) indexing plate, (5) pulley block, (6) weights, (7) base.

The common calibration method for medium range multi-axis force sensors [27–30] are utilized. A series of standard input forces in three directions are loaded on the sensor separately, and output voltages in all three directions are recorded simultaneously. The diagram of the static calibration experiment setup is shown in Figure 4, which consists of a loading plate, a calibration shaft, a 3-axis force sensor, an indexing plate, a pulley block, weights and a base.

**Figure 5.**
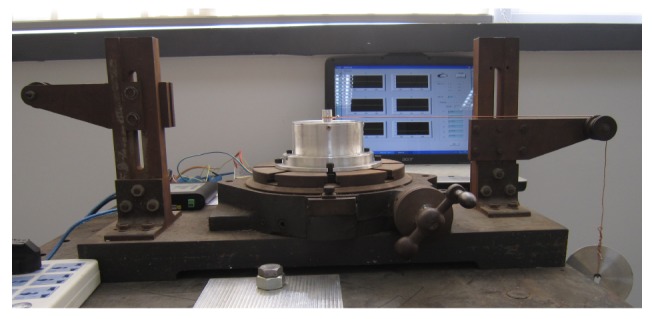
The static calibration experiment setup.

The force sensor is fixed on a horizontally rotatable indexing plate with scale to ensure the direction of loading forces. The maximum error of the weights is ±5 mg. Loading forces in X direction and Y direction are generated by pulley block and weights while indexing plate is rotated by laboratory technicians to ensure the loading direction. During force loading process in Z direction, a series of standard weights are put directly on the loading plate. All loading forces are transferred from weights to force sensors through the calibration shaft. The experiment platform of static calibration experiment is shown in Figure 5.

The calibration range is from −100 N to +100 N with an increment step of 10 N in X direction and Y direction. The calibration range in Z direction is from −150 N to 0 N with an increment step of 5 N. The whole calibration experiment is carefully repeated six times. Six sets of calibration data are obtained and zero-corrected. No gross errors are tested in the zero-corrected data.

## 4. Results

### 4.1. Decoupling by a Standard RBF

Cross validation is used to estimate the performance of decoupling by a standard MIMO RBF NN. The cross validation is repeated three times while each two sets of the zero-corrected calibration, named as “test data I” and “test data II”, are used for verification and the other four sets are used as training data. Matlab Neural Network Toolbox is used for simulations. The variance is set to 1, the maximum MSE error tolerance is set to 2.4 × 10^−6^.

The maximum MSE error tolerance is chosen to be small enough to ensure that all training data be selected as centers of the MIMO RBF NN.

In order to test the decoupling accuracy, interference errors *γ* are calculated. Taking calibration data in X direction for example, the interference error *γ_x_* is calculated in Equation (12),

(12) $$\gamma_{x}=\frac{\max\left|f_{y(m)}\right|+\max\left|f_{z(m)}\right|}{f_{x(FS)}}$$

where *f_x_*_(_*_FS_*_)_ denotes the full scale value of loaded force in X direction. The interference error *γ_x_* accounts for maximum coupling errors in Y direction and Z direction induced by loaded force in X direction. *γ_y_* and *γ_z_* are calculated in the same way. Interference errors of initial data and data after decoupling by a standard MIMO RBF NN are shown in Table 1.

Table 1 shows that the decoupling algorithm by a standard MIMO RBF is unreliable, because although some interference errors are reduced to less than 1%, some interference errors are even worse than initial data (in boldface). This is because compared with the number of training data, there are too many parameters in the MIMO RBF NN. Also, different coupling error elements are not well related. For example, there is little relationship between *e_xy_* and *e_xz_*. However, decoupling by a standard MIMO RBF NN means all the relationships are regarded as the same in the training process. The standard MIMO RBF NN does not have a good conformability with the calibration data shape. As a result, random noise in the training data may easily contribute to overfitting.

### 4.2. Decoupling by the Coupling Error Model and *ε*-SVR

**Figure 6.**
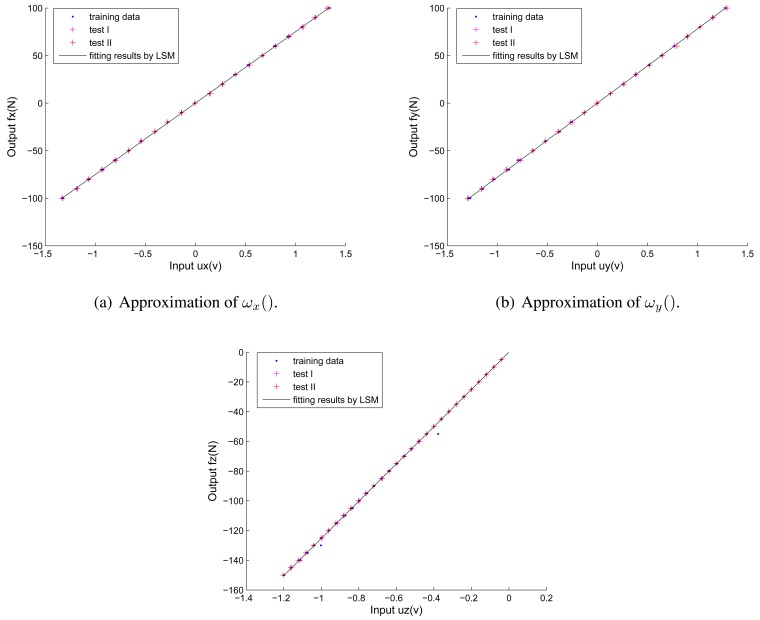
Linear fittings of non-coupling functions.

We also use cross validation to test the performance of our proposed decoupling algorithm. Here we take the first round of cross validation for example. To quantify the strong linearity of non-coupling functions, linear fittings are performed on the training data (plotted as blue points) to determine the slopes and strength of the correlations (plotted as black lines) as shown in Figure 6.

The linear correlation coefficients for all three directions are calculated to be more than 0.99, which correspond to assumption 1 (see Section 2). The inverse functions of non-coupling functions are obtained as:

(13) $$\{\begin{array}{l} f_{x}=\omega_{x}^{-1}(u_{x})=75.065u_{x}\\ f_{y}=\omega_{y}^{-1}(u_{y})=77.788u_{y}\\ f_{z}=\omega_{z}^{-1}(u_{z})=125.17u_{z} \end{array}$$

The six corrected coupling functions are approximated by *ε*-SVRs. Each corrected coupling function is fitted by a separate *ε*-SVR, where the pre-specified parameters are set as the same value. The *ε*-SVR based approximations of corrected coupling functions are carried out using MATLAB and LIBSVM [31] software applications. LIBSVM [31] is an integrated software for support vector classification, distribution estimation and regression. It implements an SMO-type optimization algorithm [32] and provides an efficient interface for MATLAB. The initial parameters used to train the *ε*-SVR model by LIBSVM tool are listed in Table 2.

Here we also employ six separate Single Input Single Output(SISO) RBF NNs to do nonlinear approximations of the corrected coupling error functions to make a comparison with *ε*-SVRs. Parameters of the SISO RBF NNs are defined the same as the MIMO RBF NN, which is mentioned in Section 4.1.

**Figure 7.**
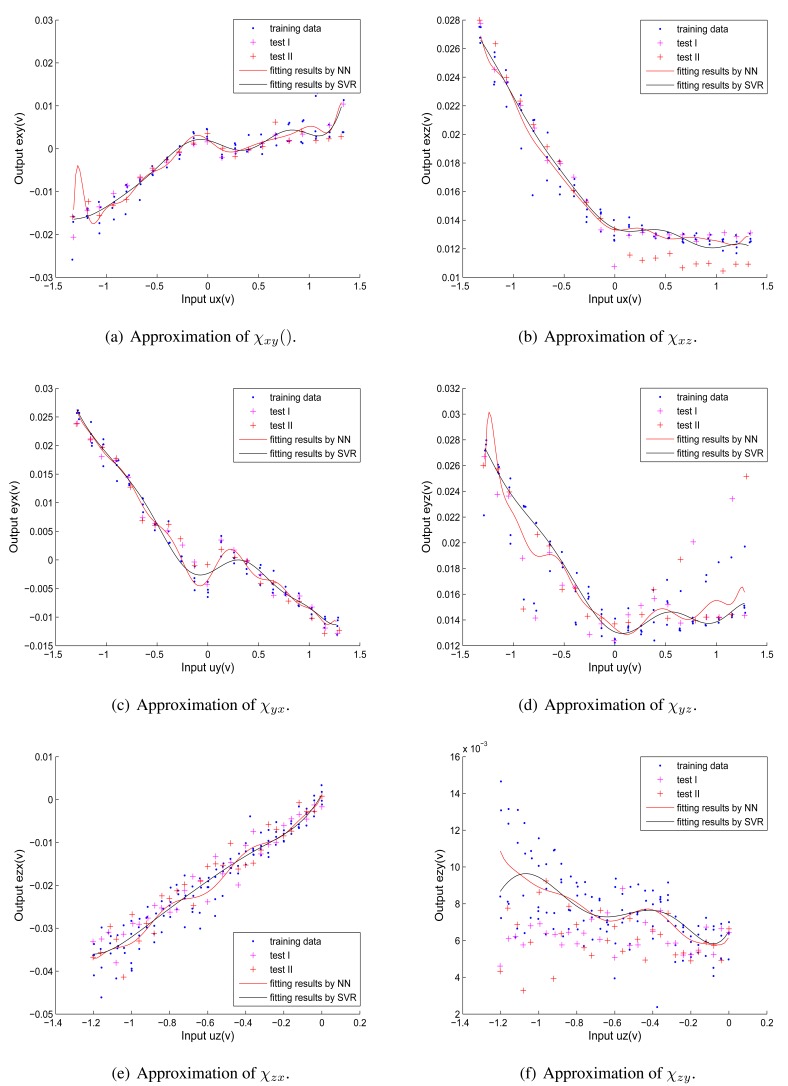
Approximation of corrected coupling functions by *ε*-SVR *vs.* by RBF NN.

Figure 7 shows the fitting results of the six corrected coupling functions.The blue points represent training data, the red points and magenta points represent two groups of test data respectively, the black curves represent fitted corrected coupling functions using *ε*-SVR and the red curves represent fitted corrected coupling functions using SISO RBF NN. Relationships between disturbing forces and related coupling error elements are nonlinear and distinct from one another, which correspond to assumption 2 and assumption 3 (see Section 2.1). In Figures 7(a) and 7(d), obvious overfittings happen in the fitting results of *χ_xy_* and *χ_yz_* using SISO RBF NNs, but the *ε*-SVR provides a good generalization ability to approximate different kinds of nonlinear functions without overfitting.

In the same fashion, the other two rounds of cross validation are calculated and interference errors are listed in Table 3. In Table 3, all interference errors are reduced to less than 1.6% FS. after decoupling by *ε*-SVR. Compared with Table 1, the decoupling accuracy is much more stable because no overfitting happens. Therefore, the proposed decoupling algorithm based on our coupling error model and *ε*-SVR is more robust.

### 4.3. Processing Time

Besides decoupling accuracy, another crucial criterion for a successful decoupling algorithm is the processing speed of both model establishment and decoupling an output voltage vector in an actual force perception task, especially the latter for on-line task purposes. To evaluate the processing speed, the elapsed time of the above two stages is recorded and the whole process is repeated 10 times. All calculations are done on a Windows XP Inter(R) Xeon(TM) 2 QUAD CPU, 3.0 GHz processor with 3.00 GB RAM. An overview of the averaged elapsed time is shown in Table 4. Table 4 shows that the elapsed time of the proposed algorithm in both the model establishment and the decoupling of an output voltage vector is less than decoupling by a standard RBF NN. *ε*-SVR proves to be fast regressions. The elapsed time of decoupling a certain output voltage vector using a coupling error model based on *ε*-SVR is less than 0.001 s. This means that the decoupling frequency is higher than 1,000 Hz. The speed of testing process based on *ε*-SVR is fast.

Summarizing, the calculation results of the decoupling methods demonstrate that our decoupling method based on our coupling error model and *ε*-SVRs has high reliability and fast running speed when no gross errors exist in the calibration data. In the next section, we will artificially add gross errors to the calibration data and analyze the decoupling method's robustness to gross errors.

### 4.4. Robustness to Gross Errors

Now we simulate the decoupling algorithm's reaction to gross errors. The gross errors in calibration data are also called *outliers*. As the quantity of gross errors in calibration data is always small, in our research, only one gross error is generated in one dimension while the calibration data of other dimensions remain the same. Here we take the first round of cross validation for example. The decoupling process is the same as Section 4.2. We artificially generate a gross error into the calibration data in X direction to test the gross error's effect on the decoupling accuracy in all dimensions. The gross error is added artificially by arbitrarily moving a point away from its initial location.

**Figure 8.**
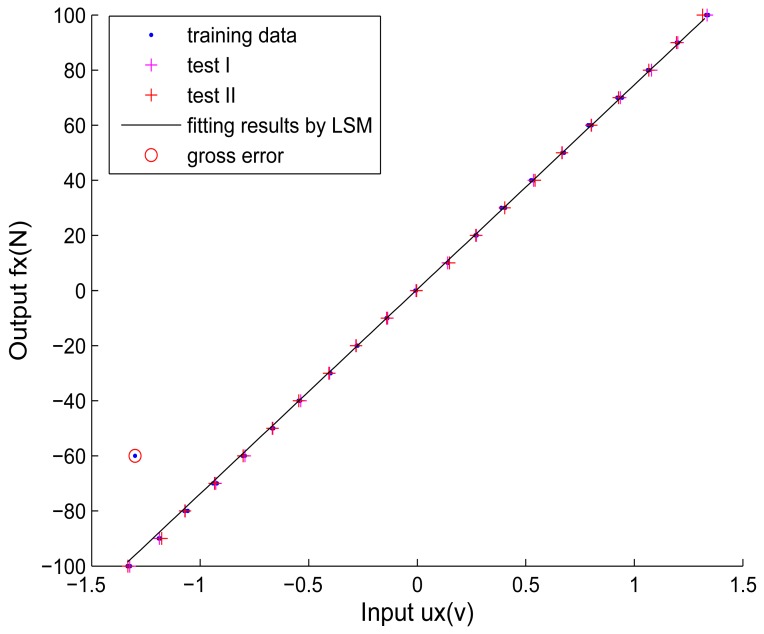
The fitting result of the *ω_x_* for calibration data with gross error.

**Figure 9.**
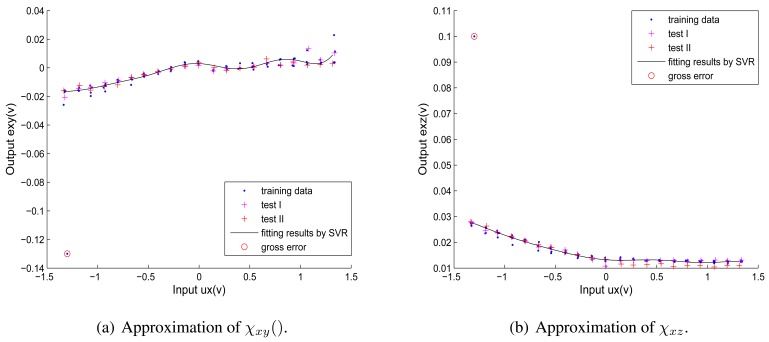
The fitting results of *χ_xy_*() and *χ_xz_* for calibration data with the gross error.

As no gross errors are introduced into the calibration data of other dimensions, the fitting results of functions in Y direction and Z direction will remain the same as shown in Figures 6 and 7. The fitting results of the non-coupling function *ω_x_*_()_ and corrected-coupling functions *χ_xy_*() and *χ_xz_*() in X direction with the gross error (plotted with red circles) are shown in Figures 8 and 9. The inverse function of *ω_x_*_()_ is obtained as:

(14) $$f_{x}=\omega_{x}^{-1}(u_{x})=74.193u_{x}$$

Comparing Figure 9 with Figure 7, when the obtained calibration data contain a gross error (outlier), the *ε*-SVR is not sensitive to the gross error and performs with high reliability, which correspond to the analysis in Section 2.2.2. Consequently, the proposed decoupling algorithm based on *ε*-SVR displays a strong robustness to the gross error. The calculated interference errors of test data I and test data II under the gross error's effect are listed in Table 5. Comparing Table 5 with Table 3, when the gross error is artificially introduced, *γ_x_*, *γ_y_* and *γ_z_* after decoupling by *ε*-SVR of both test data I and test data II seem almost unchanged. The comparison of interference errors also confirms that *ε*-SVR is robust to gross errors.

## 5. Conclusions

In this paper, a robust decoupling algorithm to efficiently reduce coupling errors for 3-axis force sensors is presented. The decoupling algorithm is based on the establishment of a novel coupling error model and *ε*-SVR. In the coupling error model, input forces are partitioned into prime forces and disturbing forces, and the corresponding output voltages are partitioned into prime voltages and coupling error elements. The structure of the coupling error model makes the decoupling process in one dimension separated from other dimensions. Linear relationships between prime forces and prime voltages in every dimension are fitted by LSM. Nonlinear relationships between disturbing forces and corresponding coupling error elements are fitted by *ε*-SVR.

The experimental results show the effectiveness of the decoupling method. All interference errors are reduced to less than 1.6%. Compared with decoupling algorithm using one standard MIMO RBF NN, the proposed decoupling algorithm demonstrates a better tolerance to noises and faster calculation speed (with the decoupling frequency higher than 1,000 Hz). It also shows robustness to gross errors. As a result, compared with existing decoupling methods, the decoupling algorithm based on the proposed coupling error model and *ε*-SVR is much more reliable in complex scenarios.

## Figures and Tables

**Table 1.** Error analysis of initial data and decoupling by a standard MIMO RBF.

		*γ_x_*		*γ_y_*		*γ_z_*	

		initial data	decoupled by RBF	initial data	decoupled by RBF	initial data	decoupled by RBF
the first	test I	2.54%	0.42%	**2.57%**	**3.73%**	2.36%	0.17%
cross-validation	test II	2.37%	0.61%	**2.52%**	**7.99%**	2.55%	0.23%
the second	test I	2.31%	1.02%	2.59%	2.32%	2.98%	0.71%
cross-validation	test II	**2.73%**	**3.45%**	2.65%	0.74%	2.81%	0.69%
the third	test I	2.40%	0.49%	2.68%	0.31%	2.57%	0.34%
cross-validation	test II	2.32%	0.26%	2.71%	0.31%	2.14%	0.32%

**Table 2.** Initial parameters used by the LIBSVM train tool.

**Parameter**	**Value**
SVM type	3(*ε*-SVR)
Kernel function	2(RBF)
*δ*^2^ (Standard deviation of kernel function)	1
C (Regularization parameter)	1000
*ε* (Deviation)	0.0001
Tolerance	0.001

**Table 3.** Error analysis of decoupling algorithm by *ε*-SVRs.

		*γ_x_*	*γ_y_*	*γ_z_*
the first	test I	1.69%	1.33%	0.82%
cross-validation	test II	1.45%	1.28%	0.59%
the second	test I	1.30%	1.47%	0.86%
cross-validation	test II	1.56%	1.24%	0.76%
the third	test I	1.43%	1.52%	0.67%
cross-validation	test II	1.48%	1.57%	0.83%

**Table 4.** Mean elapsed time (in seconds) of the training process and the testing process.

	**a RBF NN**	*ε***-SVRs**
Establishment of the model	80.5603	3.3702
Decoupling of an output voltage vector	0.1404	0.00093

**Table 5.** Error analysis of decoupling by *ε*-SVRs when a gross error is introduced.

		*γ_x_*	*γ_y_*	*γ_z_*
the first	test I	1.41%	1.51%	0.71%
cross-validation	test II	1.44%	1.56%	0.87%

## References

[1] Perry D. (1997). Multi-axis force and torque sensing. Sens. Rev..

[2] Lefebvre T., Xiao J., Bruyninckx H., De Gersem G. (2005). Active compliant motion: A survey. Adv. Robot..

[3] Siciliano B., Villani L. (1999). Robot Force Control.

[4] Siciliano B., Khatib O. (2008). Force Control. Springer Handbook of Robotics.

[5] Xu G., Song A., Li H. (2011). Adaptive impedance control for upper-limb rehabilitation robot using evolutionary dynamic recurrent fuzzy neural network. J. Intell. Robot. Syst..

[6] Nagata F., Kusumoto Y., Watanabe K., Tsuda K., Yasuda K., Yokoyama K., Omoto M., Miyako H. Polishing Robot for PET Bottle Molds Using a Learning-Based Hybrid Position/Force Controller.

[7] Lee S., Asada H. (1999). A perturbation/correlation method for force guided robot assembly. IEEE Trans. Robot. Autom..

[8] Song A., Wu J., Qin G., Huang W. (2007). A novel self-decoupled four degree-of-freedom wrist force/torque sensor. Measurement.

[9] Beyeler F., Muntwyler S., Nelson B. Design and Calibration of a Microfabricated 6-Axis Force-Torque Sensor for Microrobotic Applications.

[10] Kim K., Sun Y., Voyles R., Nelson B. (2007). Calibration of multi-axis MEMS force sensors using the shape-from-motion method. IEEE Sens. J..

[11] Oddo C., Valdastri P., Beccai L., Roccella S., Carrozza M., Dario P. (2007). Investigation on calibration methods for multi-axis, linear and redundant force sensors. Meas. Sci. Technol..

[12] Voyles R., Morrow J., Khosla P. Shape from Motion Approach to Rapid and Precise Force/Torque Sensor Calibration.

[13] Cao H., Yu Y., Ge Y. A Research of Multi-Axis Force Sensor Static Decoupling Method Based on Neural Network.

[14] Lei J., Qiu L., Liu M., Song Q., Ge Y. Application of Neural Network to Nonlinear Static Decoupling of Robot Wrist Force Sensor.

[15] Ming D., Zhang X., Liu X., Wan B., Hu Y., Luk K. Nonlinear Static Decoupling of Six-Dimension Force Sensor for Walker Dynamometer System Based on Artificial Neural Network.

[16] Cristianini N., Shawe-Taylor J. (2000). An Introduction to Support Vector Machines: And Other Kernel-Based Learning Methods.

[17] Guo J., He Y., Liu C. (2011). Nonlinear correction of photoelectric displacement sensor based on least square support vector machine. J. Cent. S. Univ. Technol..

[18] Wang X. Non-Linearity Estimation and Temperature Compensation of Capacitor Pressure Sensors Using Least Square Support Vector Regression.

[19] Smola A., Schölkopf B. (2004). A tutorial on support vector regression. Stat. Comput..

[20] Vapnik V. (1998). Statistical Learning Theory.

[21] Verrelst J., Muñoz J., Alonso L., Delegido J., Rivera J., Camps-Valls G., Moreno J. (2012). Machine learning regression algorithms for biophysical parameter retrieval: Opportunities for sentinel-2 and-3. Remote Sens. Environ..

[22] Wang L. (2005). Support Vector Machines: Theory and Applications.

[23] Platt J.C. (1998). Fast Training of Support Vector Machines Using Sequential Minimal Optimization. Advances in Kernel Methods: Support Vector Machines.

[24] Dahai L., Tianshi L. On-Line Robust Modeling of Nonlinear Systems Using Support Vector Regression.

[25] Chuang C., Su S., Jeng J., Hsiao C. (2002). Robust support vector regression networks for function approximation with outliers. IEEE Trans. Neural Netw..

[26] Ma J., Song A. (2012). Development of a novel two-axis force sensor for chinese massage robot. Appl. Mech. Mater..

[27] Berkelman P., Whitcomb L., Taylor R., Jensen P. (2003). A miniature microsurgical instrument tip force sensor for enhanced force feedback during robot-assisted manipulation. IEEE Trans. Robot. Autom..

[28] Chao L., Yin C. (1999). The six-component force sensor for measuring the loading of the feet in locomotion. Mater. Des..

[29] Chen L., Song A. A Novel Three Degree-of-Freedom Force Sensor.

[30] Xu K., Li C., Zhu Z. (2007). Dynamic modeling and compensation of robot six-axis wrist force/torque sensor. IEEE Trans. Instrum. Meas..

[31] Chang C.C., Lin C.J. (2011). LIBSVM: A library for support vector machines. ACM Trans. Intell. Syst. Technol..

[32] Fan R., Chen P., Lin C. (2005). Working set selection using second order information for training support vector machines. J. Mach. Learn. Res..

